# Robot-assisted subretinal injection system: development and preliminary verification

**DOI:** 10.1186/s12886-022-02720-4

**Published:** 2022-12-12

**Authors:** Kunkun Yang, Xin Jin, Zhaodong Wang, Yifan Fang, Zhao Li, Zhe Yang, Jinju Cong, Yang Yang, Yifei Huang, Liqiang Wang

**Affiliations:** 1 Graduate School of Chinese PLA General Hospital, 100853 Beijing, China; 2 Senior Department of Ophthalmology, the Third Medical Center, Chinese PLA General Hospital, 100039 Beijing, China; 3 School of Mechanical Engineering and Automation, Beihang University, 100191 Beijing, China; 4 School of Medicine, Nankai University, 300071 Tianjin, China; 5 Aier Eye Hospital, 433199 Qianjiang City, Hubei Province China; 6 State Key Laboratory of Kidney Diseases, 100853 Beijing, China

**Keywords:** Subretinal injection, Medical robot, Ophthalmic robot, Robot-assisted surgery

## Abstract

**Background:**

To design and develop a surgical robot capable of assisting subretinal injection.

**Methods:**

A remote center of motion (RCM) mechanical design and a master-slave teleoperation were used to develop and manufacture the assisted subretinal surgery robot (RASR). Ten fresh isolated porcine eyes were divided into the Robot Manipulation (RM) group and Manual Manipulation (MM) group (5 eyes for each group), and subretinal injections were performed by the robot and manual manipulation methods, respectively. A preliminary verification of the robot was performed by comparing the advantages and disadvantages of the robot manipulation and manual manipulation by using optical coherent tomography (OCT), fundus photography, and video motion capture analysis after the surgery.

**Results:**

Both the robot and the manual manipulation were able to perform subretinal injections with a 100% success rate. The OCT results showed that the average subretinal area was 1.548 mm^2^ and 1.461 mm^2^ in the RM and MM groups, respectively (*P* > 0.05). Meanwhile the volume of subretinal fluid obtained using the retinal map mode built in OCT was not statistically different between the RM and MM groups (*P* > 0.05). By analyzing the surgical video using Kinovea, a motion capture and analysis software, the results suggest that the mean tremor amplitude of the RM group was 0.3681 pixels (x direction), which was significantly reduced compared to 18.8779 pixels (x direction) in the MM group (*P* < 0.0001).

**Conclusion:**

Robot-assisted subretinal injection system (RASR) is able to finish subretinal injection surgery with better stability and less fatigue than manual manipulation.

## Introduction

As a novel intraocular drug delivery method that can act directly on retinal tissues, subretinal injection has been increasingly used in scientific research and clinical practice in ophthalmology, especially for submacular hemorrhage (SMH), gene and stem cell therapy [1, 2]. Different from intravitreal injections, gene and stem cell therapy has more demanding requirements in terms of distance of action and residence time of the drug, aiming to maximize the effect while minimizing the amount of drug used. This is why the application of intravitreal injections, which allow the drug to diffuse within the vitreous cavity and eventually target the retina, has been constrained in these new therapeutic approaches. However, subretinal injections, unlike intravitreal injections, are increasingly popular, as they allow for precise intraocular targeting, have a direct effect on retinal tissue, minimize intraocular diffusion of the drug, and simultaneously increase the duration of interaction with the retina [1–3].

Notwithstanding these advantages, subretinal injections are more delicate to perform and put forward a higher requirement for ophthalmologist’s operation. Contrary to intravitreal injections, which can be done under surface anesthesia, subretinal injections allow the puncture needle to be inserted precisely between the photoreceptor layer and the retinal pigment epithelium (RPE) under local or general anesthesia and inject the drug while maintaining good stability to avoid further damage to the retina [4, 5]. Still, many scholars are exploring this area, for example, the use and modification of existing devices to achieve the operation of subretinal injection. In a study of Shi et al., 2022, the investigators modified a 1 ml syringe and connected it to the viscous fluid control unit of a vitrectomy device to enable practical clinical application of subretinal injection as a therapy for SMH [6]. But this homemade device is still inadequate in terms of stability and safety, so a special device that can maintain stability during the operation is especially important.

Robotic technology can help the surgeon to improve the precision and stability of the surgery with its own advantages and thus perform more complex surgeries, and considering that ophthalmic surgery is mostly performed under microscopic vision, robotic technology combined with microscopic technology can theoretically help the ophthalmologist to perform more delicate surgical operations while reducing secondary injuries during surgery [7, 8]. Edwards et al. first demonstrated the feasibility of using robot-assisted intraocular surgery on humans, with significantly fewer secondary injuries resulting from robot-assisted intraocular surgery compared to traditional manual procedures [9]. Gijbels et al. performed the world’s first retinal vein vascular thrombolysis with the assistance of a self-developed robotic system [10], and other scholars have also completed strabismus correction and cataract surgery under the aid of a robotic surgical system [11, 12]. The tremble in robot-assisted retinal surgery can be effectively eliminated, and the movement of the end instrument in the eye can be decreased, thus reducing secondary injuries [13, 14]. Furthermore, the ophthalmic robot can be equipped with intraoperative OCT and other devices that can observe the position and posture of intraocular instruments in real time and give feedback immediately, which increases the safety of the surgical procedure [15]. Therefore, the application of a robotic surgical system to retinal injection surgery appears to be a feasible approach that can improve surgical precision and mitigate secondary injuries.

While the iRAM!S and Preceyes ophthalmic surgical robots are capable of performing surgical operations with subretinal injections [16, 17], and there are few such studies in China [18]. So, in this study, we designed and constructed an ophthalmic surgical robotic system with high stability, which aims to be capable of performing subretinal injection operations, and the performance of the system was initially verified in isolated porcine eye model.

## Methods

### Robot system design and construction

Classical medical robots such as the Da Vinci still are able to perform the external ocular surgery, but in more delicate intraocular surgery, the structure and size of the system itself limits its performance while offering no advantages in terms of operative time and effect [11]. Then, some scholars proposed a method that combine the remote center of motion (RCM) theory and the human-machine cooperative operation concept to meet the motion and operation of the robot system for intraocular surgery, and developed the steady-hand eye robot system (SHER) finally [19]. The mechanical design of the RCM constrains the end of the instrument well within the eye, while the master-slave teleoperation makes the procedure more intuitive and facilitates shorter learning time for the ophthalmologist.

**Fig. 1:**
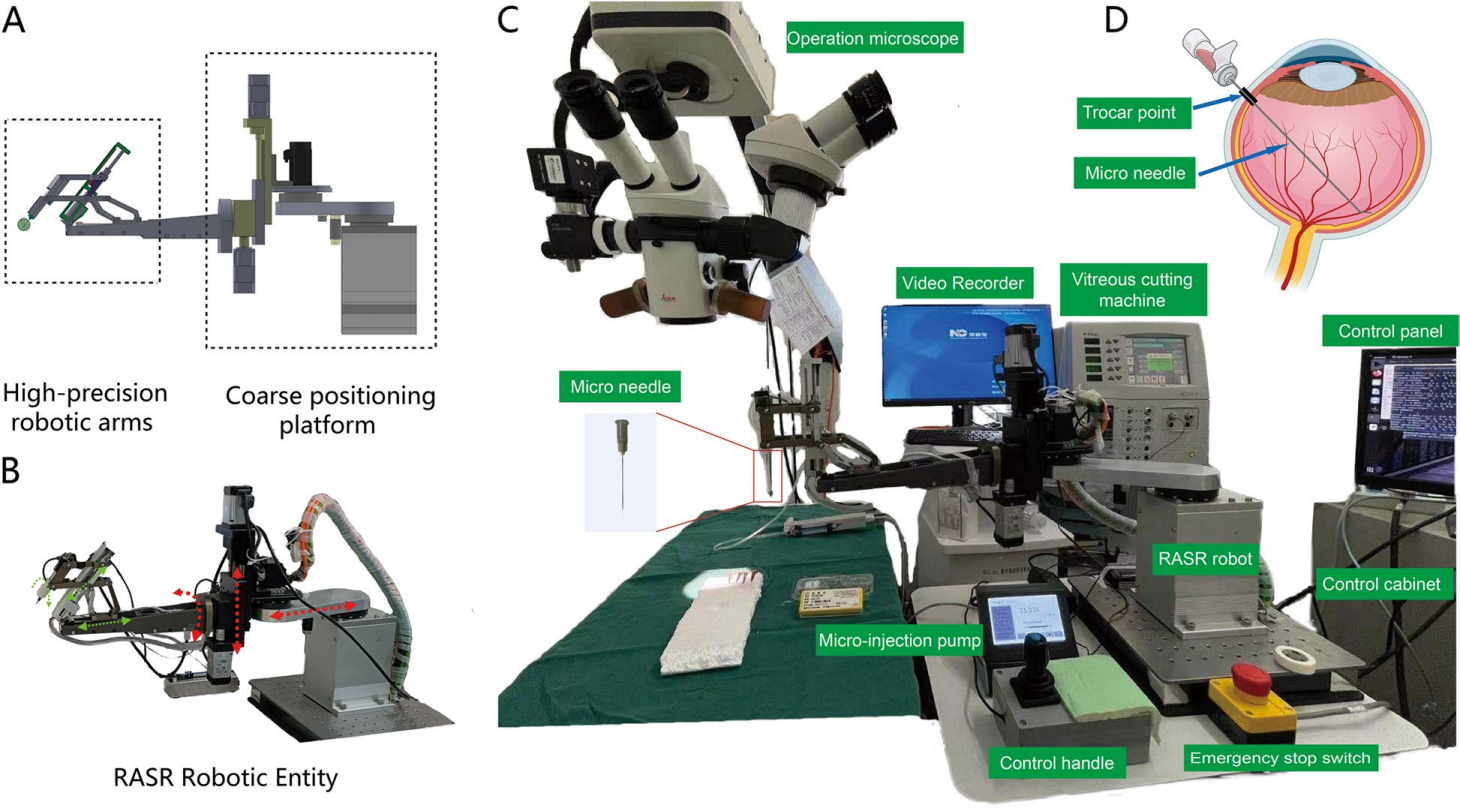
The experimental environment and the equipment and devices used in the study. **A** Schematic diagram of the RASR robot body. The robot consists of a coarse positioning platform and a high-precision robotic arm with a parallelogram-based RCM configuration, which allows the surgical instruments to move around the puncture point; **B** Photograph of the RASR machine. The red and green arrows respectively represent the direction in which the coarse positioning stage and the high precision robot arm can have rotation; **C** the equipment and devices used in the study, mainly including: operation microscope (Leica, Germany), vitreous cutting machine (Cirrus, Alcon, America), Video recorder (NCVideo, Niukangman, China), Micro-injection pump (QSI-53,311, Stoelting, America), Micro needle (41G, INCYTO, Korea) and control panel, control cabinet, control handle (joystick) and emergency stop switch for RASR robot; **D** Schematic procedure of subretinal injection: establish the flat pars plana vitrectomy channel, deliver the micro needle into the eye through the scleral trocar point, and select the appropriate site for piercing under microscopic guidance

Cooperated with the Beihang University (BUAA, China), we designed and developed the robot-assisted subretinal injection robot (RASR), which consists of two mutually perpendicular rotational joints and one planar joint (Fig. 1). Each rotating joint contains two sliding pairs driven by linear motors, and the rotation is achieved by adjusting the movement of the sliding pairs. During the procedure, the precise location of the surgical instruments and the rotation around the virtual RCM point are achieved by precisely adjusting the angle and movement of the two rotating joints. And the flat joint is mounted on the bottom of the robot body to hold the mechanical arm and to achieve the feeding motion of the surgical instruments. The robot consists of a coarse positioning platform and a high-precision robotic arm with a parallelogram-based RCM configuration that allows the surgical instruments to move around the puncture point. The coarse positioning stage enables fast and efficient attitude adjustment of the robot arm, while the high precision robot arm can theoretically achieve an end position accuracy of 14 μm, 10 μm and 4 μm in X-axis, Y-axis and Z-axis respectively. However, the theoretical value of position accuracy has not been verified by experiment.

The robot uses a joystick as the master hand and a robot containing an RCM mechanism as the slave hand, coupled with an ophthalmic microscope to provide visual feedback to the surgeon. The positive and negative kinematic model of the robot was analyzed, and the kinematic mapping relationship between the master hand and slave hand of the robot was established to solve the problems of scleral constrained movement, accurate positioning of the injection position, stabilization of the instrument during injection, and micro-flow injection during this intraocular operation.

### Experimental environment and surgical procedures

In this experiment, we used fresh isolated porcine eyes for subretinal injection and divided 10 eyes into Robot Manipulation (RM) group and Manual Manipulation (MM) group (5 eyes for each group) according to the different manipulation methods. The porcine eyes used in our experiments were purchased from slaughterhouses (Hengkangjian company, Beijing, China). And none of the authors were involved in the handling of the pigs or the acquisition of the porcine eyes. The RASR robot is located on the temporal side of the fresh pig-eye model and is operated by means of a joystick. Currently, Linux terminals are used to capture the joystick signals and transmit them to the robot control system for manipulation.

**Fig. 2:**
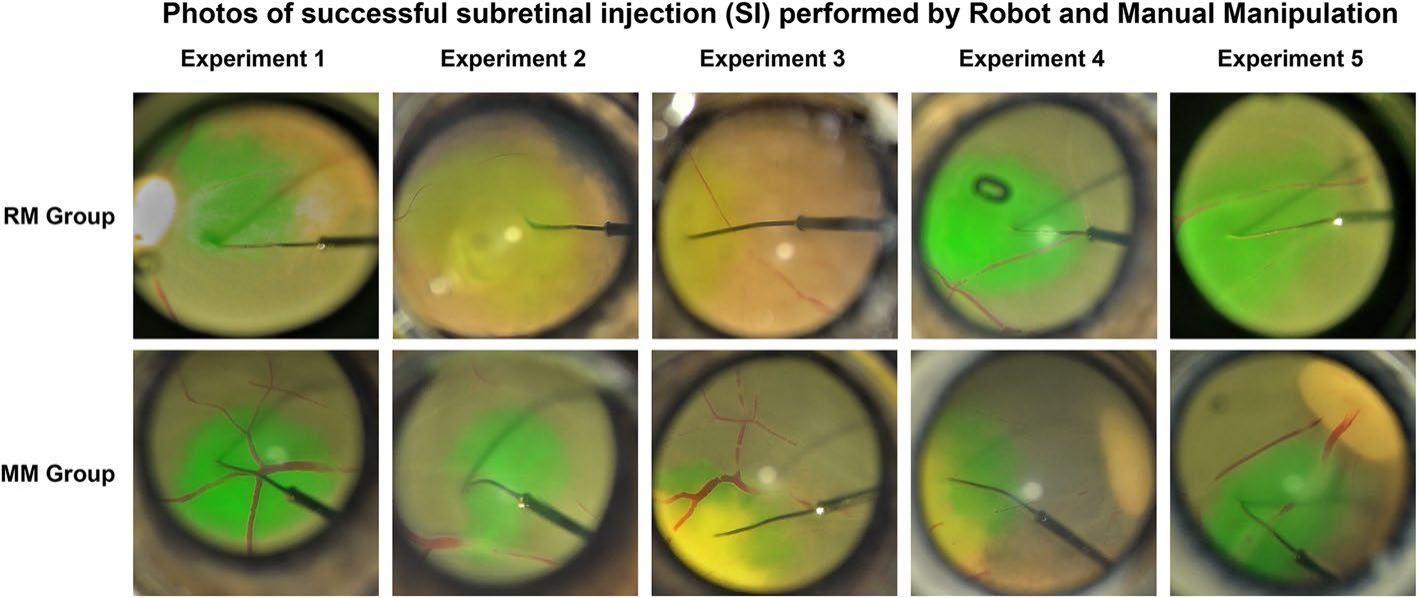
The funds photography of successful subretinal injection in RM and MM group. Successful subretinal injection requires the following conditions, including entry of the puncture needle tip into the retina, formation of a superficial retinal bulge after injection of simulated fluid, absence of extensive retinal detachment, and no outflow seen in the vitreous cavity

The surgery setting and the instrument needed are shown in Fig. 2, and the surgical procedures (RM group) are as follows.

- Check the retina of the porcine eye to ensure that the retina of the fundus is on site, otherwise it will be replaced, then place the porcine eye on the eye table.
- Adopt the conventional planar vitrectomy approach, establish a triple channel for planar vitrectomy, and establish intraocular irrigation connected to the inferior temporal channel, then connect the intraocular penetrating micro needle (41G, INCYTO, Korea) to the micro-injection pump (QSI-53,311, Stoelting, America), and drain the air in the connecting tube.
- Initialize the position of the high-precision robotic arm, load the 41G intraocular puncture needle into the designated position at the end of the high-precision robotic arm, and place the RASR robot on the temporal side of the pig eye model.
- The lead surgeon manipulates the control rocker to adjust the position of the robot’s coarse positioning platform to allow the high-precision robotic arm and the end intraocular puncture needle to approach the superior temporal scleral puncture tunnel, and then continues to adjust the angle of the high-precision robotic arm to align the direction of needle entry with the direction of the scleral tunnel. The high precision robotic arm is then manipulated to insert the needle in the direction of the scleral tunnel, aligning the virtual RCM point with the scleral puncture point and completing the registration of the RCM point.
- The assistant surgeon guides the intraocular illumination fiber through the supra-nasal scleral puncture tunnel into the eye, turns on the surgical microscope video system, displays the live image, adjusts the microscope focus until the surgical video image is clear, and examines the posterior pole of the retina under the intraocular illumination fiber. The lead surgeon selects the puncture position through the surgical video system and then starts the surgical video recording.
- The lead surgeon continues to control the 41G intraocular puncture needle slowly into the eye through the superior temporal scleral puncture channel, and when the needle appears in the field of view, the position of the arm is manipulated to adjust the position of the needle to avoid injury to the lens and retina.
- The surgeon continues to control the joystick to make the robotic arm take the 41G intraocular puncture needle slowly into the eye through the supratentorial scleral puncture channel, and when the needle appears in the field of view, the position of the arm is controlled to adjust the position of the needle to the selected puncture position. Slowly control the movement of the puncture needle to avoid injury to the lens and retina.
- After the lead surgeon has controlled the robotic arm to the designated position via the joystick, he then proceeds to control the robotic arm to perform the puncturing action of the intraocular puncture needle, allowing the tip of the puncture needle to enter under the retina (Stop operating the joystick as soon as the lead surgeon observes that the puncture needle tip has fully entered the retina). The assistant surgeon activates the microinjector and slowly injects the simulated fluid (1% sodium fluorescein) at a rate of 25 µl/min for 30 s. The lead surgeon observes the formation of the retinal bleb and leakage from vitreous cavity (defluxing effect) while the injection is being made. After the injection is completed, the lead surgeon operates the control joystick to remove the puncture needle slowly from the puncture hole and continues to observe fluid leakage (defluxing effect).

The procedure in the MM group is simpler than that in the RM group, with the lead surgeon performing the subretinal injection procedure with the hands [5].

### Video recording and OCT test

Each experiment in the RM and MM groups was recorded using a microscopic video recording system (NCVideo, Niukangman, China) for the subretinal injection procedure and stored in mpeg format. Optical coherence optical tomography (OCT) test was performed after the completion of subretinal injection in each experiment in the RM and MM groups by using an OCT instrument (OCT-HS100, Canon, Japan). Use the built-in One Line, Radial Line and Retinal Map Model to examine the pig eyes and select images with good quality control and good signal strength for preservation.

### Data processing and statistical analysis

In this study, we analyzed the surgical videos of each experiment through Kinovea (Version 0.9.5), a motion capture and analysis software, and the obtained data were tested by a non-parametric test of two independent samples (Mann-Whitney test). The height of the retinal bulge and the subretinal fluid volume in the porcine eyes were also measured by using the measurement tool available with the OCT instrument.

## Result

**Fig. 3:**
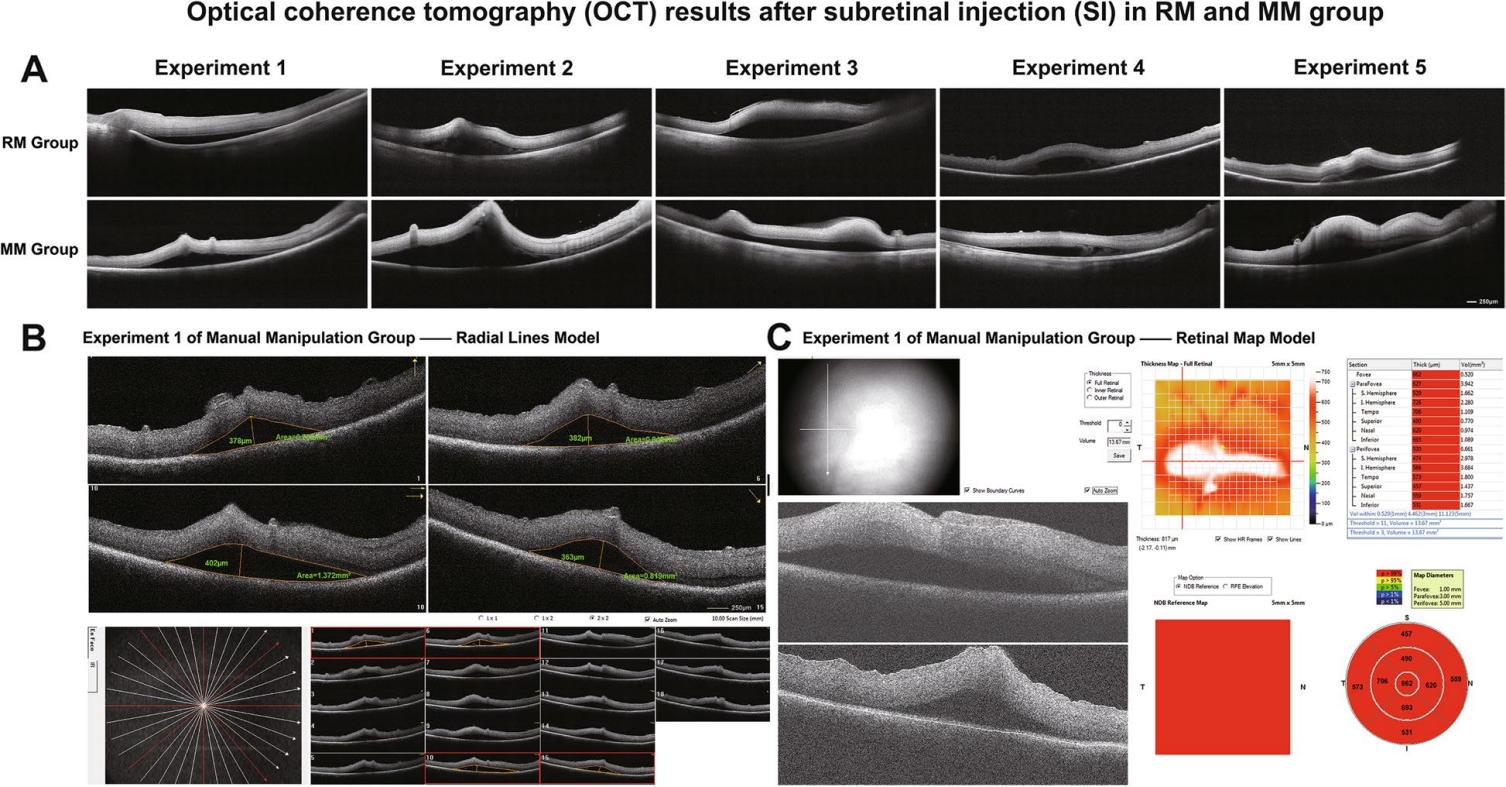
Optical coherence tomography test and results of retina after subretinal injection. **A** The retinal images acquired with the one line model clearly showed a slight retinal bulge and good closure of the piercing hole in the RM and MM groups; **B** The retinal bulge was observed and analyzed from different angles by circumferential scanning of the piercing site with the radial line model, and the cross-sectional area of the retinal bulge was measured on four OCT images selected from different quadrants; **C** The volume of the subretinal fluid was measured using the retinal map model

We recorded the surgery video of each experiment in the Robot Manipulation Group and the Manual Manipulation Group, and selected pictures of the subretinal injections (Fig. 2). The criteria for a successful subretinal injection are: (a) the tip of the puncture needle enters the retina, (b) superficial retinal bulge is observed after the injection of simulated fluid, (c) no extensive retinal detachment occurs, and (d) no outflow was seen in the vitreous cavity. OCT examination was performed immediately in each porcine eye model to evaluate the retinal bulge in the fundus when subretinal injection surgery was done. And the results of each trial in RM and MM group suggested mild retinal bulge without retinal tears or extensive detachment. By fundus photography and retinal OCT, both the RM and MM groups were shown to have a 100% success rate of subretinal injection. After that, we acquired 16 images from different angles on the area of the subretinal injection by using the Radial Line Model of the OCT instrument (Fig. 3B). Four different quadrants of OCT images (1, 6, 10, 15) were chosen to measure the subretinal sectional area (Area 1, Area 6, Area 10, Area 15), and the average sectional area was calculated to approximate the subretinal volume. The results showed that the average subretinal area was 1.548 mm^2^ and 1.461 mm^2^ in the RM and MM groups, respectively, with no statistically significant differences between the two groups (*P* > 0.0 5, Table 1). Meanwhile the volume of subretinal fluid obtained using the Retinal map mode built in OCT (Fig. 3 C) was not statistically different between the RM and MM groups (*P* > 0.05, Table 1).

**Table 1:** Measurement and analysis of retinal cross-sectional area and subretinal fluid volume, and no differences were seen between the RM and MM groups. (*P* > 0.05, Independent sample t-test)

Group		Area 1 (mm^2^)	Area 6 (mm^2^)	Area10 (mm^2^)	Area15 (mm^2^)	Average Area (mm^2^)	Retinal map volume (mm^3^)
Robot Manipulation Group	Experiment 1	1.037	1.179	1.012	1.279	1.127	15.670
	Experiment 2	1.258	1.419	1.707	1.142	1.382	14.510
	Experiment 3	4.182	3.928	3.034	3.531	3.669	10.060
	Experiment 4	1.153	1.082	1.197	1.179	1.153	13.810
	Experiment 5	0.584	1.012	1.488	0.607	0.923	14.790
Manual Manipulation Group	Experiment 1	0.766	0.849	1.372	0.819	0.952	13.670
	Experiment 2	3.878	4.033	3.812	3.896	3.905	8.740
	Experiment 3	0.656	0.922	1.042	0.669	0.822	14.000
	Experiment 4	0.801	1.230	1.486	0.885	1.101	15.340
	Experiment 5	1.973	2.053	0.996	1.034	1.514	15.150
						*P* > 0.05	*P* > 0.05

**Fig. 4:**
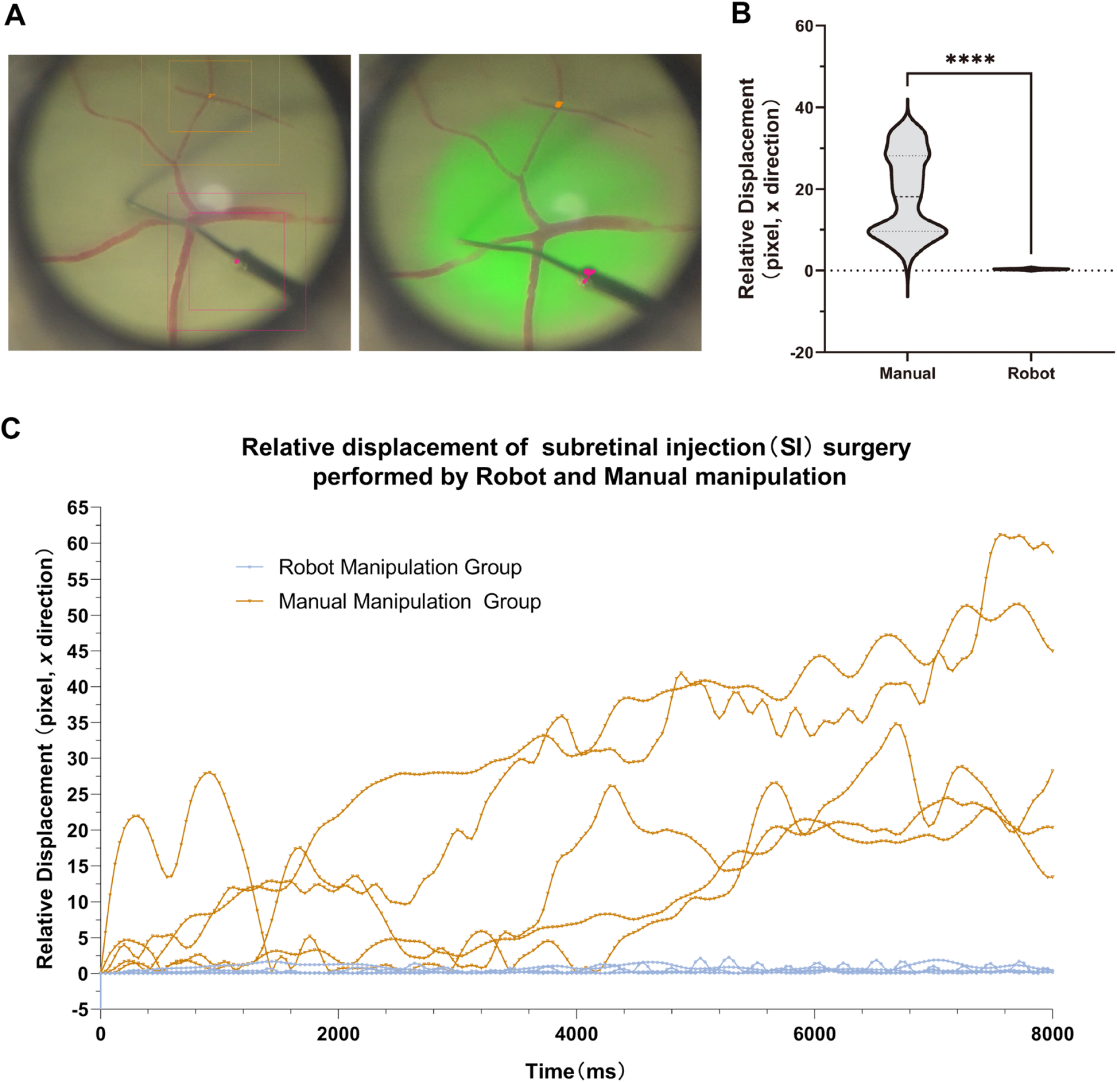
Capture and analysis of relative displacement of end instruments. **A** The acquisition interface of Kinovea video motion capture software, the orange point on the left is the baseline point, recording the displacement of the eye itself, the orange box is the tracking Windows, the inner is the object window while the outer is the search window. The red point is the record point, which records the displacement change of the end instrument; the orange and red parts of the right picture are the motion trajectory of the baseline point and record point respectively, while the absolute value of the difference between the baseline point and the record point is used as the relative displacement value; **B** Violin plot of relative displacement (Median, Interquartile Range, IQR), and a significant decrease in relative displacement was observed in the RM group compared to the MM group (*p* < 0.0001); **C** The line graphs of relative displacement with time for each experiment in the RM and MM groups show that the RM group remained stable over the entire time, while the MM group showed an increase in relative displacement with time

To investigate the stability of both robotic and manual manipulation, we used Kinovea software to track a frame by frame and analyze the tremor of the surgical operation. Considering that the micro needle has two different temporal phases in the eye: motion and rest, we first intercepted the video of micro needle at rest after completing the piercing action in each experiment, then we selected the middle point of the micro needle as the recording point of the tremor amplitude of the micro needle (Fig. 4 A, red point), meanwhile, we also chose the intersection of the peripheral retinal vessels as the baseline point of the tremor amplitude of the eye (Fig. 4 A, orange point). Finally, by subtracting the data of the baseline point from the data of the recording point, we obtained the relative motion amplitude of the micro needle. The mean motion amplitude of RM and MM groups were 0.3681pixels and 18.8779pixels (x direction), and the motion amplitude of RM group was significantly reduced compared with that of MM group (*P* < 0.0001, Fig. 4B). It can also be seen that the motion amplitude of the MM group increased obviously with the prolongation of the operation time, while the RM group maintained a good stability.

To further compare the operative time between robot-assisted (RM group) and conventional surgery (MM group), we used surgical videos in this study to count the operative time in the RM and MM groups (taking into account the difference in preoperative preparation time between the two manipulations, this study starts with the entry of the puncture needle from the scleral tunnel into the eye and ends with the completion of the retinal puncture). The results showed that the mean operative time in the RM group was 254.4s compared to 82.2s in the MM group, with the RM group taking longer than the MM group (*p* = 0.0159, by independent samples t-test) (Table 2).

**Table 2:** Procedures time (s) in RM and MM groups

	Experiment 1	Experiment 2	Experiment 3	Experiment 4	Experiment 5	average procedure time	*p* Value
RM group	269	278	230	285	210	254.4	0.0159
MM group	104	80	79	78	70	82.2	

## Discussion

In this study, the RASR, an assisted subretinal injection surgical robot, was designed and manufactured, and its function was preliminarily validated. The results of surgical video recording, fundus photography, and OCT examination indicated that both robot and manual manipulation were able to perform subretinal injection in both the RM and MM groups, and no significant differences were observed in postoperative vitreous fluid, retinal augmentation, and subretinal volume between the groups. These results suggest that the RASR robot was able to control the movement of the micro needle within the eye, get to the designated location and eventually complete the subretinal injection procedure without any complication.

Kinovea, a video analysis software, was used to analyze the surgical video frame by frame and obtain relative tremor amplitude to quantify the surgical video content. During the experiment, we found that human-caused tremors were transmitted to the porcine eye fixation device and the surgical microscope, although such tremors were very slight. In addition, to improve the recognition accuracy of Kinovea during the video processing, we selected the intersection of blood vessels of retina far from the subretinal piercing area as the recording point (baseline point) during the process, and on the other hand, we picked the midpoint of the micro needle as the recording point, considering that the tip of the penetrating needle (micro needle) could not be recognized by the software when it entered the subretinal. The results suggest that the RASR robotic operation in the RM group showed less tremor during the subretinal injection procedure compared with the manual operation in the MM group (*P* < 0.0001), and other than that, we also observed that the end instrument tremor gradually increased in the MM group as the operation time was extended, which was not observed in the RM group (Fig. 4). The above results show that the RASR robot can greatly reduce intraoperative tremors, and at the same time, it can overcome the physiological characteristics of manual fatigue and maintain the stability of surgical instruments for a long time. The results of Noda, Cutler et al. also support that the tremor of the end instrument is significantly reduced under robot-assisted conditions and is able to overcome the fatigue effect of human hands [20, 21].

We operated OCT instrument to scan the porcine eye model after subretinal injection surgery, and achieved accurate imagines of the retina after surgery by using high-definition tomography (one line) method, which could show the retinal bulge and the size of the retinal tears better than fundus photographs. However, it is impossible to observe all aspects of the subretinal injection area in that scanning mode, so we chose the Radial Line mode in the OCT instrument to acquire retinal images that were used for observation of the piercing hole and measurement of retinal detachment. The OCT results suggested that the height of retinal bulge and subretinal fluid volume were not statistically different after the subretinal injection procedure completed by the two operations, but the high retinal bulge in Experiment 2 of the MM group can be seen in Fig. 3 A, which does not exclude the risk of retinal detachment, even though no obvious retinal fissures were detected.

Regrettably, the intraoperative OCT device was not used in this study because of the prevalence of COVID-19. Although the intraoperative OCT device still has shortcomings in terms of scan depth, scan speed and image clarity, it can provide real-time feedback to the surgeon, especially in extremely delicate retinal procedures which are beyond the surgeon’s perception. Although its imaging quality is still not sufficient to measure the volume of subretinal fluid, the miniaturized OCT probe developed by Abid et al. is a significant improvement over conventional OCT devices in terms of measurement accuracy and scanning speed [22]. Meanwhile, scholars have also used machine learning techniques to develop a real-time tool for real-time stratified distance estimation using intraoperative 4D OCT for robotic subretinal injections [23]. We have always believed that the introduction of intraoperative OCT devices would be essential for ophthalmic surgical robots, enabling an extended range of perception for physicians. Collaboration with OCT manufacturers and teams in medical imaging and artificial intelligence is currently underway to explore this area further.

The increasing popularity of subretinal injections with the rise of stem cell therapy and gene therapy is inextricably linked to the unique advantages of subretinal injections, which are mainly reflected in the ability of subretinal injections as a drug delivery method to act directly on the target area while reducing the amount of drug used and doing no damage to the structure of the retina [24]. Considering that intravitreal injections may lead to off-target effects and other side effects, subretinal injections can work directly on the retina and utilize the anatomy of the eye to form a local bulge that can constrain the drug on the target for a long time and effect. In addition, the intraocular micro needle used for subretinal injection is mostly 41G with an internal diameter of about 90 μm, while the physiological tremor in normal people is in the range of 50–200 μm. So, it needs to be kept stable for fine operations such as retinal piercing and drug infusion, otherwise it may cause enlargement of the retinal tears or even intravitreal fluid leakage, and even in rare cases it may cause serious complications such as detachment of the retina. When an enlarged retinal tear or even intravitreal fluid leakage is detected during the subretinal injection procedure, a planar vitrectomy and intraocular laser photocoagulation are required to close the retinal tears to ensure safety, which not only increases the operative time and lowers the treatment outcome, but also reduces the patient’s benefit-to-risk ratio. The 41G needle used for subretinal injection is theoretically capable of avoiding the use of intraocular laser photocoagulation to close the retina tears as long as the device can be kept stable enough. The RASR robot provided a highly stable surgical operating environment in this study, avoiding intraocular photocoagulation, and no postoperative examinations, including OCT, showed retinal tears.

However, it is inevitable that subretinal injection surgery still carries some risk of retinal detachment and other risks, so it is essential to maintain the stability of the surgical instruments. Additionally, Scruggs et al. indicated that high flow rates during subretinal drug infusion can cause damage to the structure of the RPE [25], and it is relatively safe to maintain a low flow rate during drug infusion, but this can increase hand fatigue and end-device instability for a manual approach. For the characteristics of subretinal injection surgery, high precision and high stability of surgical operation are the basis to ensure the safety and decline the complications of the surgery. However, these conditions cannot be completely met by manual manipulation, while the RASR robot from this study is capable of providing such an environment. The RASR robot can minimize end-instrument tremor and provide a precise and stable surgical environment, allowing for better protection against the aforementioned complications and improving the patient benefit-to-risk ratio.

In our experiments, we found that although the manual manipulation was faster than the RASR robot in entering and exiting the needle, it was difficult to maintain stability for long periods of time during the piercing and drug infusion process, whereas the robot-controlled piercing needle performed significantly better than the manual manipulation in maintaining stability throughout the procedure. The average procedure time in the RM group in this study was 254.4 s, while the average procedure time in the MM group was 82.2 s. As we performed the experimental validation of the RASR robot, we also found some difficulties and aspects of the system that deserve optimization. For example, the virtual RCM point coinciding with the scleral piercing point does not dominate in terms of time efficiency, which makes the procedure time for robot-assisted subretinal injection longer than that for manual operation, in line with the conclusions reached in other studies [17, 26]. Another thing is that the surgical path planning of the RASR robotic system is not yet perfect, which also increases the time of robotic surgery.

The surgical methods and scope of intraocular surgery has been greatly expanded since the introduction of planar vitrectomy by Machemer et al [27], but the level of sophistication and complexity of vitreoretinal surgery has also increased due to the complex structure and soft tissues and narrow space of the human eye. At the same time, physical hand tremor is an unavoidable physiological phenomenon that can negatively affect microsurgery, while robot-assisted conditions can effectively filter tremor, improve surgical precision and stability, and decline tissue damage, without increasing its learning time compared to conventional vitreoretinal surgery [13, 28]. The RASR surgical robot is capable of performing retinal injections, but we also hope to realize more extensive fundus surgical operations, such as vitrectomy and anterior macular membrane peeling, by replacing the end instruments, and to realize a semi-automatic or even fully automatic RASR surgical robot by combining artificial intelligence recognition, intraoperative OCT tracing, and reasonable surgical path planning.

## Conclusion

In conclusion, we designed and developed a surgical robot to assist subretinal injection in this study, which was validated in a fresh isolated porcine eye model and evaluated for results using OCT and video analysis. The results suggest that that the robot is able to finish subretinal injection surgery with better stability and less fatigue than manual manipulation, which lays the foundation for the next improvement and realization of more functions.

## Data Availability

The raw datasets supporting the conclusions of this article are available from the corresponding author Dr. Liqiang Wang with the email: liqiangw301@163.com.

## Abbreviations

RASR: Robot-assisted subretinal injection system
RCM: Remote center of motion
RM: Robot Manipulation
MM: Manual Manipulation
OCT: Optical coherent tomography
SMH: Submacular hemorrhage
RPE: Retinal pigment epithelium
SHER: Steady-hand eye robot system
DOF: Degree of freedom
